# Transcranial photobiomodulation add-on therapy to valproic acid for pentylenetetrazole-induced seizures in peripubertal rats

**DOI:** 10.1186/s12906-022-03562-9

**Published:** 2022-03-21

**Authors:** Chung-Min Tsai, Shwu-Fen Chang, Hsi Chang

**Affiliations:** 1 Graduate Institute of Medical Sciences, College of Medicine, Taipei Medical University, Taipei, Taiwan; 2 Department of Pediatrics, MacKay Children’s Hospital, Taipei, Taiwan; 3 Department of Pediatrics, School of Medicine, College of Medicine, Taipei Medical University, Taipei, Taiwan; 4 Department of Pediatrics, Taipei Medical University Hospital, 250 Wuxing St., Taipei, 11031 Taiwan

**Keywords:** Transcranial photobiomodulation, Add-on therapy, Valproic acid, Pentylenetetrazole, Seizures, Epilepsy, Status epilepticus

## Abstract

**Background:**

Convulsive status epilepticus (CSE) prevention is critical for pediatric patients with epilepsy. Immediate intervention before CSE reduce severity. Despite its wide usage as an anticonvulsant, valproic acid (VPA) results in harmful side effects such as dose-dependent hepatotoxicity. Hence, reducing VPA dosage to minimize side effects while maintaining its efficacy is necessary, and transcranial photobiomodulation (tPBM) add-on therapy could facilitate this. We recently demonstrated for the first time that tPBM at a wavelength of 808 nm attenuated CSE in peripubertal rats. However, the effects of VPA with the add-on therapy of tPBM prior to seizures have not yet been explored. This study investigated whether adding tPBM to VPA exerts synergistic effect for CSE prevention in peripubertal rats.

**Methods:**

A gallium-aluminum-arsenide laser (wavelength of 808 nm with an exposure duration of 100 s and irradiance of 1.333 W/cm^2^ at the target) was applied transcranially 30 min after VPA injection in Sprague Dawley rats. All the rats received 90 mg/kg of pentylenetetrazole (PTZ). Except for the saline (*n* = 3), tPBM + saline (*n* = 3), and PTZ group (*n* = 6), all the rats received a PTZ injection 30 min after VPA injection. The rats received add-on tPBM with PTZ immediately after tPBM. In the VPA + PTZ group, the rats received low-dose (100 mg/kg, *n* = 6), medium-dose (200 mg/kg, *n* = 6), and high-dose (400 mg/kg, *n* = 7) VPA. In the VPA + tPBM + PTZ group, the rats received low (100 mg/kg, *n* = 5), medium (200 mg/kg, *n* = 6), and high (400 mg/kg, *n* = 3) doses of VPA. Seizures were evaluated according to the revised Racine’s scale in a non-blinded manner.

**Results:**

Adding tPBM to low-dose VPA reduced the incidence of severe status epilepticus and significantly delayed the latency to stage 2 seizures. However, adding tPBM to high-dose VPA increased the maximum seizure stage, prolonged the duration of stage 4–7 seizures, and shortened the latency to stage 6 seizures.

**Conclusions:**

Adding tPBM to low-dose VPA exerted a synergistic prevention effect on PTZ-induced seizures, whereas adding tPBM to high-dose VPA offset the attenuation effect.

## Background

Convulsive status epilepticus (CSE) is the most common childhood neurological emergency, with a mortality rate of 1% in the United States, 3–5% in the United Kingdom, and 7% in Taiwan [1–3]. Preventing CSE can reduce the severity and morbidity of pediatric patients. Current strategies for preventing CSE includes the use of closed-loop detection–treatment systems that combine the prediction, detection, and immediate treatment of CSE [4]. The US Food and Drug Administration–approved responsive neurostimulation (RNS) system (NeuroPace), a closed-loop detection treatment system [5, 6], could substantially reduce focal-onset seizures and improve patients’ quality of life [7]. Our previous study [8] suggested that the application of transcranial photobiomodulation (tPBM) prior to a CSE event—determined on the basis of a deep learning model’s prediction of seizures [9]—can alleviate CSE. Neurostimulation is applied to the closed-loop detection–treatment systems by using electricity at present, tPBM can also be used for future neurostimulation after seizure prediction and detection. The regular use of antiepileptic drugs (AEDs) for CSE prevention is crucial. With seizure prediction and the regular use of AEDs established as fundamental for CSE prevention, we evaluated whether tPBM as an add-on to routinely used AEDs would exert a synergistic effect on CSE prevention. Valproic acid (VPA or sodium valproate; trade name: Depakine) is one of the first-choice AEDs for benzodiazepine (BZD)-refractory CSE [10, 11]. Despite its frequent clinical usage, VPA may have side effects such as hyperammonemia [12] and liver toxicity (LiverTox category A) [13]. Hepatotoxicity is the most severe side effect of VPA [14]. VPA enhances hepatotoxicity by inhibiting mitochondrial beta-oxidation and shunting into cytochrome P450–associated pathways [15]. Compared with adults, children are more susceptible to VPA-induced hepatotoxicity [16]. Moreover, VPA-induced hepatotoxicity is more severe in children and infants than in adult and has high mortality following acute liver failure [17]. Furthermore, in vitro [18] and in vivo [19] studies have revealed the hepatotoxicity of VPA is dose-dependent. Although a recent cohort study reported non-dose-dependent changes in the liver enzyme levels of pediatric patients with epilepsy, the dose-dependent hepatoxicity of VPA is observed in daily clinical practice [20]. In addition, VPA-induced hyperammonemia was reported to be dose dependent [21]. Therefore, administered doses of VPA must be reduced to minimize its side effects while maintaining or even enhancing its therapeutic effects for CSE. Such dose reduction would be beneficial for pediatric patients with CSE and may be achieved through an add-on therapy.

Photobiomodulation (PBM) therapy, previously known as low-level laser (light) therapy (LLLT) [22], involves the use of red to near-infrared (NIR) light to cause biological alterations in organisms secondary to the interactions of photons in the visible or infrared spectral region with molecules in cells or tissues [23]. PBM therapy or LLLT with red to NIR light works through the absorption of photons by mitochondrial cytochrome *c* oxidase (CcO) [24], thus accelerating and increasing ATP synthesis [8, 25] and activating numerous pathways, eventually exerting cellular protective effects including anti-inflammatory effect [26, 27]. tPBM thereby is a non-invasive approach in which NIR light penetrates through the scalp–skull–dura–brain tissue via a quantum optical–induced transparency effect [28] and subsequently exerts PBM on the neurons and glia. tPBM modulates electroencephalographic rhythms [25]; increases cerebral oxidized CcO oxygenated hemoglobin levels [29, 30], cerebral blood flow [31] and brain-derived neurotrophic factor levels; and promotes synaptogenesis [32] while inhibits neuronal apoptosis [33, 34] and reduces the reactive oxygen species level in neurons under oxidative stress [27, 35]. Clinical trials have reported that tPBM exerted therapeutic effects on patients with traumatic brain injury [36], Parkinson’s disease [37], age-related cognitive decline [38], autism spectrum disorder [39], sexual dysfunction [40], anxiety and depression [41], and fear [42]. To date, the effects of tPBM on epilepsy have been investigated only in animal studies [8, 43, 44].

The application of tPBM by using NIR lasers (at wavelengths of 808 nm and 780 nm) exerted antiepileptic effects on rats [8, 44]. Previous in vitro and in vivo PBM studies supported the results and provided insights into the underlying mechanisms. Neurochemically, PBM (810 nm) could suppress the excitotoxicity (induced by kainic acid which is an chemoconvulsant that could also trigger CSE [45]) of primary cortical neurons [35]. In their in vivo study, Ahmed et al. [46] reported that tPBM (830 nm) exerted an inhibitory effect on cortical neurotransmitters in normal rats. Radwan et al. [43] demonstrated that tPBM (830 nm) reduced the CSE-induced increase in pilocarpinized rat cortical and hippocampal amino acid neurotransmitters. Hong [47] reviewed PBM studies relevant to epilepsy. Electrophysiologically, the application of PBM (632.8 nm) induced the hyperpolarization of pyramidal cells in the rat olfactory cortex [48, 49]. Kataoka et al. [50] reported that PBM (830 nm) inhibited the transient excitation of the postsynaptic potentials in rat hippocampal slice and neural activity in the primary auditory cortex in vivo. Furthermore, the application of PBM (850 nm) to the rat visual cortex suppressed visual evoked potentials [51]. Moreover, the application of the PBM (808 nm) to the rat subthalamic nucleus predominantly evoked inhibitory responses [52]. tPBM (808 nm) exerted an antiepileptic effect by promoting the function of inhibitory GABAergic interneurons [8]. The application of PBM with NIR (an sub-division of infrared) might involve properties of the “infrared light stimulation” which enhanced the amplitude, increased the frequency, and reduced the decay time constant of the spontaneous inhibitory postsynaptic currents (sIPSCs) of cultured rat cortical neurons [53]. This indicated that infrared light enhanced presynaptic γ-amino butyric acid (GABA) release, and the modulation of sIPSCs was mediated by postsynaptic GABA_A_ receptors (GABAARs) [53]. A study using an animal model of epilepsy administered pentylenetetrazole (PTZ), a noncompetitive GABA_A_ receptor antagonist, to induce CSE in rats and observed the occurrence of neuronal apoptosis during PTZ-induced CSE [54]. PTZ-induces seizures through the noncompetitive antagonism of the GABA_A_ receptor complex [55]. A study suggested that PTZ-induced seizures indirectly activated glutamate N-methyl-D-aspartate (NMDA) receptors [56], and pretreatment with MK-801, an NMDA receptor antagonist, attenuated PTZ-induced seizures in rats [56, 57]. However, compared with tPBM alone, the combination of MK-801 and tPBM (808 nm) was less effective in increasing cerebral blood flow in mice [31], indicating that the neuroprotective effect of tPBM can be relevant to NMDA receptors. Indeed, PBM (808 nm) blocked NMDA receptors in vitro [58]. In hippocampal GABAergic interneurons, parvalbumin-positive interneurons (PV-INs) –expressed the most cytochrome c, indicating that they contained the most abundant CcO [59] and thus were highly susceptible to tPBM – strongly inhibit principal cells through hippocampal gamma oscillation [60]. However, PV-INs are vulnerable to CSE-induced apoptosis [61]. tPBM (808 nm) attenuated neuronal apoptosis in the hippocampus in neonatal rats [62], and the apoptosis of hippocampal neurons was observed in PTZ-induced seizures in peripubertal rats [63]. Taken together, these findings indicated that tPBM could enhance presynaptic GABAergic interneurons, particularly PV-INs, promote the presynaptic release of GABA and thus enhance sIPSCs while attenuating PTZ’s noncompetitive antagonism toward the GABA_A_ receptor complex, and block the NMDA receptors of postsynaptic principal cells. Thus, tPBM administered at a wavelength of 808 nm could exert an antiepileptic or anticonvulsive effect on an animal model of PTZ-induced seizures or status epilepticus (SE) in peripubertal rats.

Our previous study [8] demonstrated the effects of tPBM monotherapy on seizure behaviors and SE in a rodent model of epilepsy and elucidated the mechanisms underlying these effects. We found that the transcranial application of NIR laser irradiation (at wavelength of 808 nm) applied transcranially attenuated PTZ-induced SE by protecting hippocampal PV-INs from apoptosis based on terminal deoxynucleotidyl transferase dUTP nick end labeling (TUNEL) assay [8] and preserving the integrity of the perisomatic inhibition network of PV-INs in pyramidal neurons present in the hippocampus of peripubertal rat brains based on the result of immunofluorescences [8]. Furthermore, by performing electroencephalography, Vogel et al. observed that tPBM reduced the epileptiform discharge in rats with epilepsy [44].

The benefits of polytherapy (combination or add-on therapy) using tPBM and AEDs, such as VPA, remained unknown. Combination therapy for seizures that administered AEDs and other electromagnetic waves was first demonstrated by Lotfy et al. who reported that VPA combined with whole-body low-dose gamma irradiation (LDR) exerted synergistic effects on PTZ-induced seizures in rats [64]. However, the adverse side effects of LDR, such as DNA mutation, are concerning, particularly in the context of treating children [65].

Human [29] and animal [66] studies have demonstrated that tPBM enhanced CcO activities. Li et al. reported that VPA dose-dependently increased CcO activities [67]. Thus, the enhancement of CcO activity by both tPBM and VPA suggests synergistic effects may be realized through an add-on therapy that make use of tPBM and VPA. Furthermore, VPA ameliorated neuronal apoptosis [68] and preserved the number of PV-INs [69] in the hippocampus of epileptic rats, including in SE models. Our previous study [8] reported that tPBM inhibited the apoptosis of hippocampal neurons particularly PV-INs. Epigenetically, VPA downregulated of *Scn3a* promoter activity, thus exerting an anticonvulsant effect in vitro [70] and blocked seizure-induced aberrant neurogenesis by inhibiting histone deacetylases at the epileptic dentate gyrus in vivo [71]. Additionally, PBM could modulate epigenetic events [72, 73].

The similar mechanisms of tPBM and VPA and their anticonvulsant effects suggest the possibility of using a synergistic polytherapy approach to attenuate SE. Here, we hypothesized that combination therapy with both tPBM and VPA for pretreatment prior to an acute seizure event would exert synergistic effects to prevent CSE. We investigated the effects of add-on therapy using a combination of VPA and tPBM on PTZ-induced seizures or SE in peripubertal rats based on the premise that such add-on therapy would reduce hepatotoxicity due to reduced dosage of VPA needed.

## Methods

### Animals

All animal experiments were performed in accordance with the Animal Protection Act of the Council of Agriculture, Executive Yuan, Taiwan, and complied with the ARRIVE (Animal Research: Reporting of In Vivo Experiments) guidelines and the Basel Declaration, and the 3R concept. Moreover, the animal experiments performed in this study were approved by the Laboratory Animal Center of Taipei Medical University (approval No. LAC-2019-0237) as were those performed in our previous study [8]. A total of 45 male Sprague-Dawley rats (aged 35–41 days) purchased from BioLASCO (BioLASCO Taiwan Co., Ltd., Taipei, Taiwan) were used in the study. The grouping assignment for the 45 rats was as follows: saline (*n* = 3), tPBM + saline (*n* = 3), PTZ (*n* = 6), VPA100 + PTZ (*n* = 6), VPA200 + PTZ (*n* = 6), VPA400 + PTZ (*n* = 7), VPA100 + tPBM + PTZ (*n* = 5), VPA200 + tPBM + PTZ (*n* = 6), and VPA400 + tPBM + PTZ (*n* = 3). Because our previous study indicated that tPBM could attenuate seizures and SE [8], we did not include “tPBM + PBM” as an individual group in this study. In addition, one rat assigned to the PTZ group was excluded from data analysis because of a technical error in PTZ injection. Five rats, including three rats in the PTZ group, one rat in the VPA100 + PTZ group, and one rat in the VPA100 + tPBM + PTZ group, that died due to severe SE received transcardial perfusion immediately after death [8, 74]. The brains of these five rats were harvested and immersed in 4% paraformaldehyde, followed by serial dehydration in 20% sucrose at 4 °C overnight and subsequent preservation in 30% sucrose at 4 °C for future use. The remaining 40 rats survived after seizures or SE were kept in cages for days and were finally euthanized with 60% CO2. After the rats became unconscious following 60% CO2 inhalation, they were considered dead once their heart beats stopped; this was confirmed by palpation of the chests. The rats were exposed to CO2 for at least 5–6 min according to the guideline of the Laboratory Animal Center of Taipei Medical University.

### Experimental protocol

All the rats received an intraperitoneal injection of VPA and then were observed and recorded on video for 30 min. Thirty minutes after VPA injection, the rats received tPBM for 100 s. Immediately after tPBM, the rats were subcutaneously injected with PTZ and then observed for seizure behavioral analysis through both direct observation and video recording for 1 h. The experimental sequence arrangement that rats received PTZ immediately after tPBM was in consideration of the bioavailability of tPBM and PTZ. PBM could change neural activity of the central nervous system in rats with time scale of 250 ms [51]. Even if to examine the onset of PBM in the time scale of “minutes”, the PBM took action as at least within “10 min” after laser irradiation on the peripheral nervous system of rats [49, 75, 76]. As for PTZ, the blood oxygen level-dependent signal intensities increased within 2–4 s with peak at 15 s after PTZ injection [77]. The latency to PTZ (90 mg/kg)-induced 1st generalized tonic-clinic (GTC) seizures in rats was in the time scale of 1–5 min [78, 79]). Considering that the instant action of PBM on neural activity in time scale of milliseconds to minutes and that PTZ works on brains within seconds and the latency of 1st GTC seizures is in time scale of minutes, the PTZ was thus arranged immediately after tPBM for the most appropriate therapeutic windows of tPBM against PTZ-induced seizures.

### VPA pretreatment

VPA sodium salt (CAS number 1069-66-5, Sigma-Aldrich, USA) was freshly dissolved in saline and prepared in dose of 50, 100, and 200 mg/mL for low, medium, and high subgroups, respectively. VPA doses of 100, 200, and 400 mg/kg were considered low, medium, and high, respectively [80]. The low and medium doses of VPA do not cause hepatotoxicity, whereas a high-dose of VPA (> 300 mg/kg) can cause hepatotoxicity [19]. VPA was injected into the rats intraperitoneally 30 min before PTZ injection [81] because a total of 30 min are required to reach the maximum serum concentration of VPA (110 mg/kg, which is close to the VPA dose of 100 mg/kg) [82].

### tPBM

The settings and parameters of tPBM used in the current study were similar to those used in our previous studies [8, 83]. Briefly, a gallium–aluminum–arsenide diode laser apparatus (Transverse Industries, Taiwan) with a center wavelength of 808 nm and an average radiant power of 110 mW per laser was used in the continuous operating mode. Laser beams were collimated using collimating lenses with a lens hood height of 11 mm. The beam shape was elliptical, and the major and minor axes of the laser beam at the horizontal plane of the front of the lens hood were 3.5 and 3.0 mm, respectively. Thus, the beam spot size and irradiance at the target (scalp surface) were 0.0825 cm^2^ and approximately 1.333 W/cm^2^, respectively. The exposure duration was 100 s, which yielded a radiant exposure of approximately 133.3 J/cm^2^ and a total radiant energy of 11 J/animal. The hair on the rats’ scalps was removed on the day of the experiment by using a depilatory cream. After marking the scalp by using an Eppendorf tube (internal diameter of 12 mm) with the center located on the skin corresponding to lambda, we gently wrapped the rats’ bodies in towels and used the mark on the scalp to line the inner edge of the lens hood. We irradiated each rat in the “VPA + tPBM + PTZ” group in only one treatment session with an exposure duration of 100 s (total radiant energy of 11.00 J). To minimize handling stress, the control animals in the VPA + PTZ groups were exposed to the same handling conditions, specifically concerning hair removal, body wrapping, and restraint, as those receiving tPBM, and their scalps were also attached to the laser apparatus for 100 s but with the power switched off.

### Acute seizure induction

Acute seizures were induced by administering a subcutaneous injection of PTZ, as described in our previous study [8]. In brief, PTZ powder (Sigma-Aldrich, USA) was dissolved in saline to a concentration of 25 mg/mL and injected subcutaneously into the loose skin on the rats’ backs in a single dose of 90 mg/kg [84].

### Seizure behavioral analysis

The rats were individually held in a transparent cage (42 × 42 × 21 cm) without animal bedding. The observation and video recording period for each rat lasted for 1 h, beginning 32–35 min after VPA injection and ending 1 h after PTZ injection. The time of seizure onset was instantly recorded by the observer during experiments. Seizure behavioral analysis was carried out with a 1 h post PTZ injection. Seizure behaviors were evaluated using the method reported by Lüttjohann et al. [85] with minor modifications as described in our previous study [8]. The intensity of seizures was as follows 1, sudden behavioral arrest and/or motionless staring; 2, facial jerking with the muzzle or muzzle and eye; 3, neck jerks; 4, clonic seizures in a sitting position; 5, convulsions including clonic and/or tonic–clonic seizures while lying on the belly and/or pure tonic seizures; and 6, convulsions including clonic and/or tonic–clonic seizures while lying on the side and/or wild jumping. A generalized tonic-clonic seizure (GTCS) refers to stage 5–6 seizures. Minor modifications were made as follows: we defined animal death as stage 7, mild seizures as stages 1–2, moderate seizures as stages 3–4, and severe seizures as stages 5–7. C. M. Tsai conducted the observations (one individual, once for each rat, with immediate recording of results in the laboratory notebook; camera recording was performed simultaneously) and scored seizure events. Thus, the scoring was conducted in a non-blinded manner.

We used the definition of SE reported by Sato and Woolley [86] and adjusted it according to the aforementioned seizure staging system. The onset of SE was recognized as stage 4–7 seizures [8] persisting for at least 30 s and continuing for no more than 2 min between seizures [8, 86].

During the 60 min of seizure behavioral observation, latencies to the onset of stage 1–6 seizures were evaluated during the 30-min period after PTZ injection [87]. If the latency to the seizure stage was absent, then the latency was considered to be 30 min (1800s) [88]. For stage 7 (death), the evaluation period was extended to 75 min for a rat in the PTZ group that died at 73 min. Therefore, if latencies to stage 7 were absent in all the rats alive 60 min after PTZ injection, then the latency was considered to be 75 min (5400 s).

### Statistical analysis

Data are expressed as the mean ± standard error of mean (SEM). An unpaired Student’s *t* test was used to examine the differences between the maximum seizure stages, the total duration of stage 4–7 seizures, and the latency to the onset of stage 1–7 seizures between any two groups of interest. A *p* value of < 0.05 was considered statistically significant. All statistical analyses were performed using GraphPad Prism software (GraphPad, version 6).

## Results

### Wet-dog shaking was observed in all the rats after VPA injection

Wet-dog shaking (WDS) is a typical behavioral manifestation observed after VPA treatment [89]. All 33 rats exhibited WDS after VPA injection, indicating the effect of VPA on all rats.

### Lower incidence rate of severe SE was observed in the rats treated with tPBM add-on therapy than untreated rats with low-dose VPA

Severe SE, which eventually led to death, was noted in three of the six rats (incidence rate: 50%) in the PTZ group. Although two of the six rats (incidence rate: 33%) in the VPA100 + PTZ group developed severe SE, one of them survived. Only one of the five rats in the VPA100 + tPBM + PTZ group developed severe SE (incidence rate: 20%); this incidence rate was lower than that in the VPA100 + PTZ group. This difference was attributable to only one rat. No rats that received medium- or high-dose VPA developed severe SE (Table 1).

**Table 1:** Incidence of severe SE in each group

Dose of VPA	Saline	tPBM + Saline	PTZ	VPA + PTZ	VPA + tPBM + PTZ
Low (100 mg/kg)	0	0	50% (3/6)	33% (2/6)	20% (1/5)
Medium (200 mg/kg)				0	0
High (400 mg/kg)				0	0

Data are presented as the percentage (rat numbers with severe SE/total rat numbers in the groups)

### tPBM adding to high dose VPA significantly increased the maximum seizure stage

**Fig. 1:**
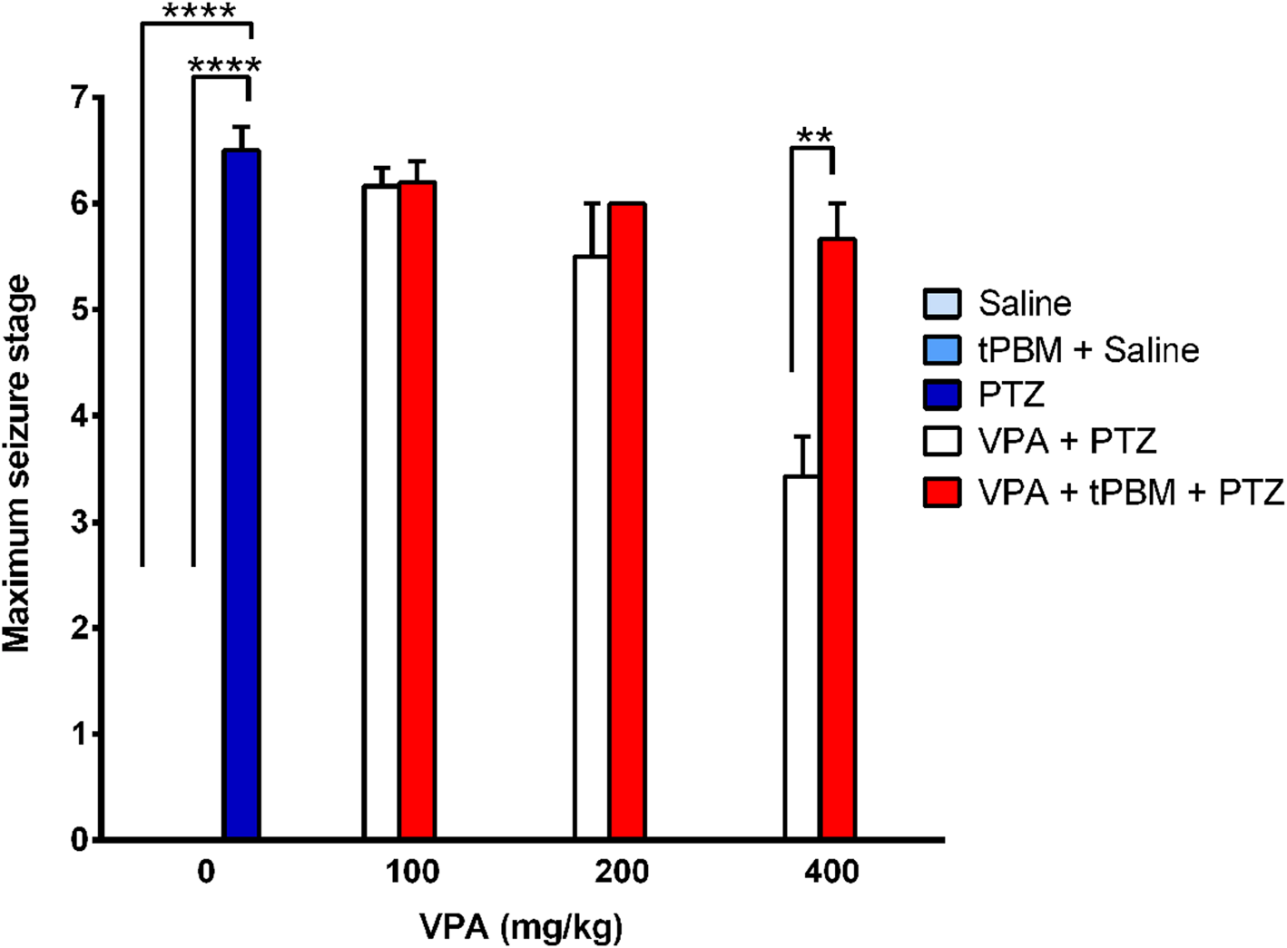
Maximum seizure stages. The maximum seizure stages in the PTZ group are shown with blue bars. The maximum seizure stages in the low-dose, medium-dose, and high-dose VPA groups are depicted as white bars. The maximum seizure stages in the rats receiving VPA and tPBM are shown in red bars. **p < 0.01; ****p < 0.0001

The maximum seizure stage in the PTZ group was 6.5 ± 0.2, which was significantly higher than that in the Saline group (stage 0, *p* < 0.0001) and tPBM + saline group (stage 0, *p* < 0.0001). When pretreated with a low-dose of VPA (100 mg/kg), the maximum seizure stages in both the VPA100 + PTZ and VPA100 + tPBM + PTZ groups were 6.2 ± 0.2. No significant difference in the maximum seizure stage was observed between the PTZ group and the rats treated with low-dose VPA. When pretreated with a medium-dose of VPA (200 mg/kg), the maximum seizure stages in the VPA200 + PTZ and VPA200 + tPBM + PTZ groups were 5.5 ± 0.5 and 6.0 ± 0, respectively. Thus, no significant difference between these two groups and between the low-dose VPA and medium-dose VPA groups with or without tPBM treatment. The maximum seizure stage that occurred in the VPA400 + PTZ group (3.4 ± 0.4) was significantly lower than that in the PTZ (6.5 ± 0.2, *p* < 0.0001) and VPA200 + PTZ groups (5.5 ± 0.5, *p* = 0.0060). However, the maximum seizure stage in the VPA400 + tPBM + PTZ group was 5.7 ± 0.3, and no significant differences in the maximum seizure stage were noted between the high-dose VPA and medium- or low-dose VPA groups among the rats receiving add-on therapy with tPBM. Furthermore, the maximum seizure stage in the VPA400 + tPBM + PTZ group was significantly higher than that in the VPA400 + PTZ group (*p* = 0.0067; Table 2 and Fig. 1). These findings indicated that add-on therapy with tPBM offsets the attenuation effect of a high dose of VPA on PTZ-induced seizures in terms of the maximum seizure stage.

**Table 2:** Maximum seizure stage and total durations of stage 4–7 seizures

	Saline	tPBM + Saline	PTZ	VPA + PTZ			VPA + tPBM + PTZ		
VPA dosage (mg/kg)	0	0	0	100	200	400	100	200	400
Maximum seizure stages	0^a^	0^a^	6.5 ± 0.2	6.2 ± 0.2	5.5 ± 0.5	3.4 ± 0.4	6.2 ± 0.2	6 ± 0	5.7 ± 0.3^b^
Total duration of stage 4–7 seizures (s)	0	0	951.3 ± 394.7	676.8 ± 417.4	29 ± 7.1	2.6 ± 1.8	566.2 ± 526.2	57.4 ± 28	35.3 ± 11.1^c^

Data are expressed as the mean ± SEM. The maximum seizure stage “0” in the tPBM + saline group indicated no seizures events

^a^significant difference (*p* < 0.0001) in respect to the maximum seizure stage in the PTZ group

^b^significant difference (*p* < 0.01) in respect to the maximum seizure stage in the high-dose VPA (400 mg/kg) + PTZ group

^c^significant difference (*p* < 0.01) in respect to the total duration of stage 4–7 seizures in the high-dose VPA (400 mg/kg) + PTZ group

### Add-on therapy of tPBM increased the total duration of stage 4–7 seizures in the high-dose VPA group

**Fig. 2:**
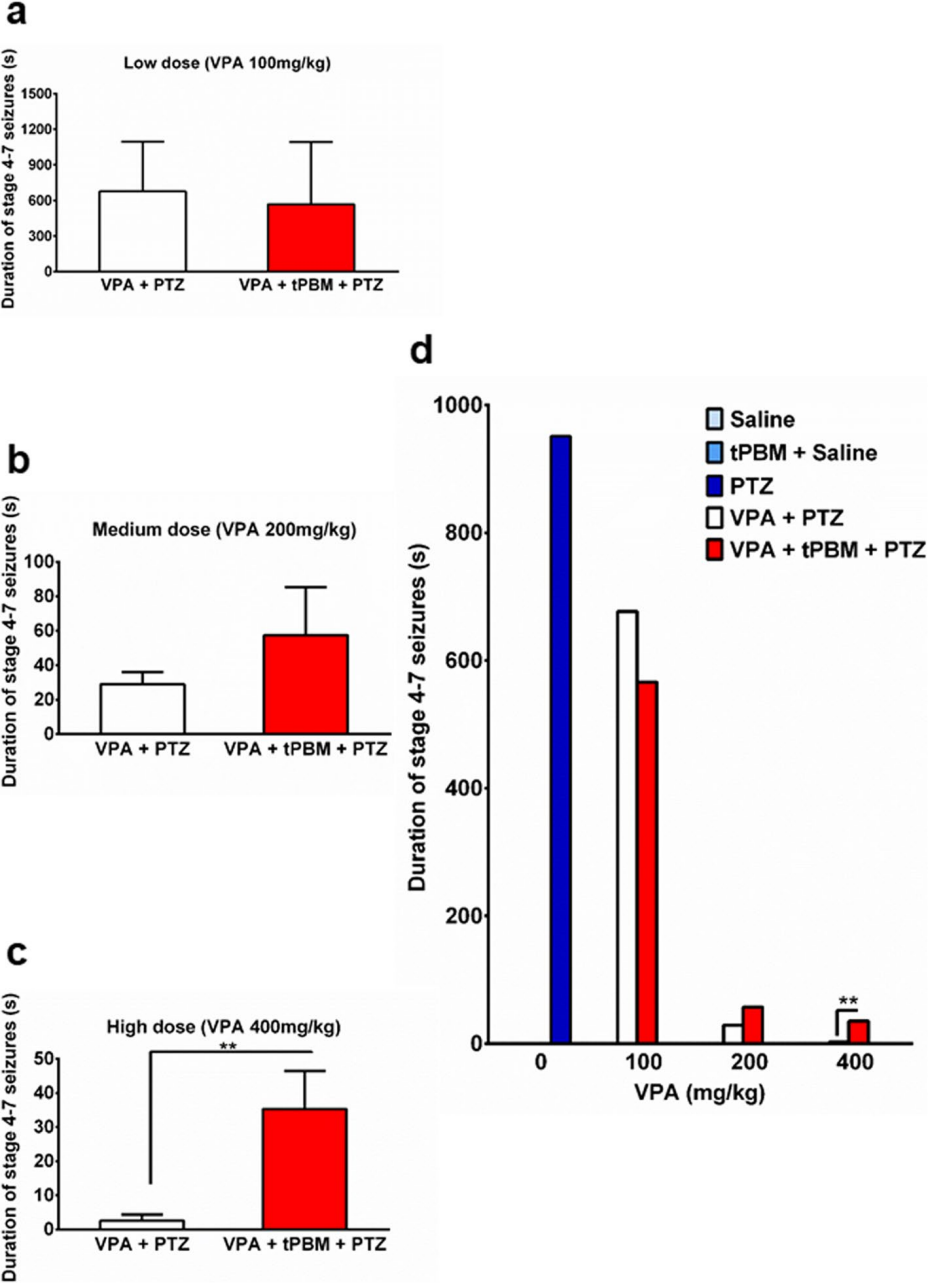
Total duration of stage 4–7 seizures. The total duration of stage 4–7 seizures in the (**a**) low-dose (100 mg/kg) VPA group (white bar) and low-dose VPA with tPBM add-on therapy (red bar); (**b**) medium-dose (200 mg/kg) VPA group (white bar) and medium-dose VPA with tPBM add-on therapy (red bar), and (**c**) high-dose VPA (400 mg/kg, white bar) and high-dose VPA with tPBM add-on therapy (red bar), **p < 0.01. **d** The total duration of stage 4–7 seizures for all the groups on the same scale (with unit of seconds). **p < 0.01

The total durations of stage 4–7 seizures were 951.3 ± 394.7 s, 676.8 ± 417.4 s, and 566.2 ± 526.2 s in the PTZ, VPA100 + PTZ, and VPA100 + tPBM + PTZ groups, respectively (Table 2, Fig. 2d). Although the mean total duration of stage 4–7 seizures with add-on tPBM was shorter than that without add-on tPBM among the rats receiving a low-dose of VPA, the differences was not significant (Table 2, Fig. 2a and d). At a medium dose of VPA, the total duration of stage 4–7 seizures in the VPA200 + tPBM + PTZ group was 29 ± 7.1 s, which was significantly shorter than that in the PTZ group (*p* = 0.0416). However, the total duration of stage 4–7 seizures in the rats receiving a medium dose of VPA with add-on tPBM (57.4 ± 28 s) was nonsignificantly longer than that in the rats receiving a medium dose of VPA without tPBM (Table 2, Fig. 2a and d). The total duration of stage 4–7 seizures in the VPA400 + PTZ group was 2.6 ± 1.8 s, which was significantly lower than that in the PTZ group (*p* = 0.0240) and VPA200 + PTZ (*p* = 0.0025) groups, respectively, and lower (but not significantly) than in the VPA100 + PTZ group (*p* = 0.1065). However, among the rats pretreated with a high dose of VPA, the total duration of stage 4–7 seizures with add-on tPBM therapy (35.3 ± 11.1 s) was significantly longer than that without add-on tPBM therapy (**p < 0.01 [*p* = 0.0018]; Table 2, Fig. 2c and d).

### Adding tPBM to low-dose VPA significantly delayed the latency to stage 2 seizures, whereas adding tPBM to high-dose VPA significantly shortened the latency to stage 6 seizures

**Fig. 3:**
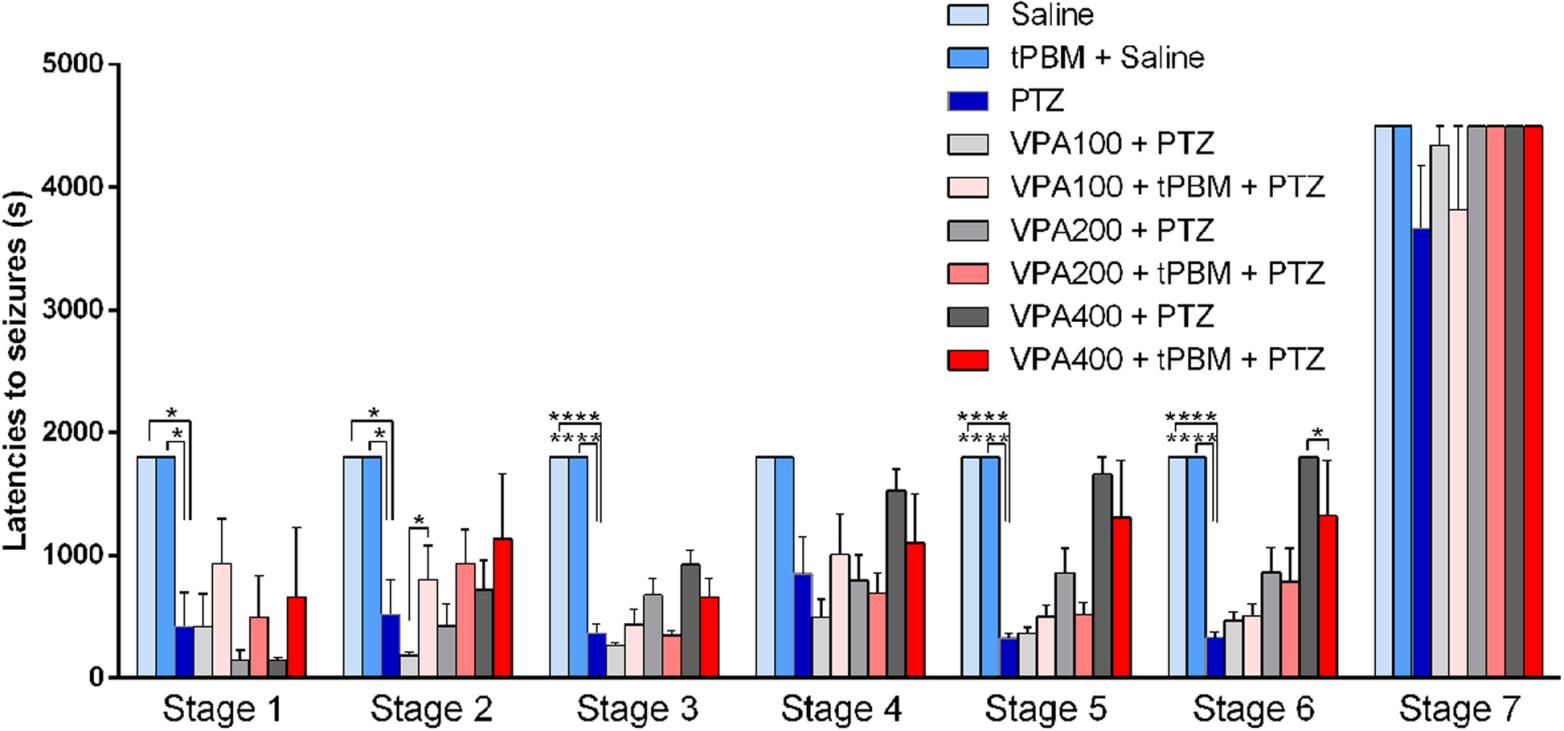
Latencies to the onset of stage 1–7 seizures. The latency to the onset of stage 1–7 seizures in the tPBM + saline group (white bar), PTZ group (blue bar), low-dose (100 mg/kg) VPA group (light-gray bar), low-dose VPA with tPBM add-on therapy (pink bar), medium-dose (200 mg/kg) VPA group (gray bar), medium-dose VPA with tPBM add-on therapy (salmon pink bar), high-dose VPA (400 mg/kg, darkslategray bar), and high-dose VPA with tPBM add-on therapy (red bar), *p < 0.05; ****p < 0.0001

The latencies to stage 1–3 and 5–6 seizures were significantly shorter in the PTZ group than in the saline and tPBM + saline groups, respectively (*p* < 0.05 in the analysis of stage 1 and 2 seizures and *p* < 0.0001 in the analysis of stage 3, 5, and 6 seizures, Table 3 and Fig. 3). However, no significant difference in the latencies to stage 4 seizures and death (stage 7) was noted between these two groups. For the rats receiving a low dose of VPA, the latency to stage 2 seizures in the VPA100 + tPBM + PTZ group (799.2 ± 281.3 s) was significantly longer than the latency to stage 2 seizures in the VPA100 + PTZ group (185.7 ± 30.93 s, *p* = 0.0402, Table 3 and Fig. 3). In terms of the latencies to stage 1 and 3–7 seizures, no significant difference was observed between these two groups. For the rats receiving a medium dose of VPA, no significant difference was observed in all the stages between the tPBM treated and untreated groups. For the rats receiving a high dose of VPA, no significant difference in stage 1–5 seizures was noted between the tPBM-treated and untreated groups. However, the latency to stage 6 seizures was significantly shorter in the VPA400 + tPBM + PTZ group (1923 ± 922.1 s) than in the VPA400 + PTZ group (3600 ± 0 s, i.e., no occurrence of stage 6 seizures, *p* = 0.0160, Table 3 and Fig. 3).

**Table 3:** Latencies to the onset of stage 1–7 seizures

Saline		tPBM + Saline	PTZ	VPA + PTZ			VPA + tPBM + PTZ		
VPA dosage (mg/kg)	0	0	0	100	200	400	100	200	400
Stage 1	1800 ± 0^a^	1800 ± 0^a^	419.0 ± 280.2	415.8 ± 272.8	146.8 ± 80.17	147.9 ± 21.78	932.6 ± 366.7	495.6 ± 333.5	659.3 ± 570.4
Stage 2	1800 ± 0^b^	1800 ± 0^b^	520.5 ± 280.4	185.7 ± 30.93	424.7 ± 180.3	716.1 ± 244.0	799.2 ± 281.3^c^	931.8 ± 282.4	1136 ± 523.0
Stage 3	1800 ± 0^d^	1800 ± 0^d^	369 ± 66.93	263.3 ± 28.31	676.2 ± 135.8	925.7 ± 121.3	433.6 ± 126.8	349.0 ± 36.93	659.3 ± 151.2
Stage 4	1800 ± 0	1800 ± 0	851.2 ± 301.1	496.5 ± 146.0	795.5 ± 208.7	1527 ± 176.5	1009 ± 326.0	691.2 ± 164.0	1102 ± 397.9
Stage 5	1800 ± 0^e^	1800 ± 0^e^	324.7 ± 41.53	367.2 ± 46.44	856.5 ± 205.3	1660 ± 140.0	498.4 ± 95.08	514.6 ± 101.0	1311 ± 463.1
Stage 6	1800 ± 0^f^	1800 ± 0^f^	330.2 ± 41.77	466.5 ± 71.5	1161 ± 494.3	3600 ± 0	505.6 ± 93.93	1145 ± 621.8	1923 ± 922.1^g^
Stage 7	5400 ± 0	5400 ± 0	3667 ± 511.1	4340 ± 160.0	5400 ± 0	5400 ± 0	3816 ± 684.0	5400 ± 0	5400 ± 0

Data are expressed as the mean ± SEM

^a^significant difference (*p* < 0.05) in respect to the latency to stage 1 seizures in the PTZ group

^b^significant difference (*p* < 0.05) in respect to the latency to stage 2 seizures in the PTZ group

^c^significant difference (*p* < 0.05) in respect to the latency to stage 2 seizures in the low-dose VPA (100 mg/kg) + PTZ group

^d^significant difference (*p* < 0.0001) in respect to the latency to stage 3 seizures in the PTZ group

^e^significant difference (*p* < 0.0001) in respect to the latency to stage 5 seizures in the PTZ group

^f^significant difference (*p* < 0.0001) in respect to the latency to stage 6 seizures in the PTZ group

^g^significant difference (*p* < 0.05) in respect to the latency to stage 6 seizures in the high-dose VPA (400 mg/kg) + PTZ group

## Discussion

The results of the present study demonstrated that the adding tPBM to low-dose VPA resulted in a beneficial synergistic effect on PTZ-induced seizures in the peripubertal rats. In addition, add-on therapy with tPBM could offset attenuation effect of a high dose of VPA on PTZ-induced seizures. A lower incidence rate of severe SE was observed in rats receiving tPBM add-on therapy than in those without tPBM treatment receiving a low dose of VPA. The add-on tPBM therapy and low-dose VPA significantly delayed the latency to stage 2 seizures. Moreover, the results revealed that add-on therapy of tPBM to high-dose VPA increased the maximum seizure stage, prolonged the total duration of stage 4–7 seizures, and shortened the latency to stage 6 seizures.

Adding tPBM to low-dose VPA significantly delayed the latency to stage 2 seizures. In view of clinical applications, reducing the dosage of VPA while maintaining or even increasing the therapeutic effect on seizures or CSE can be beneficial to patients with epilepsy because the adverse effects of VPA, such as hepatotoxicity and hyperammonemia, have been reported to be dose-dependent [20, 21]. In particular, patients with epilepsy with the mutated genotypes of the cytochrome P450 isozymes CYP2C9 and CYP2A6 are more vulnerable to VPA-induced hepatotoxicity [90]. Similarly, pediatric patients with epilepsy and who harbor the mutant alleles of CYP2C9 and CYP2A6 have a higher risk of VPA-induced hyperammonemia [91]. These patients, who presented a particularly problematic clinical scenario, may benefit from adding tPBM to low-dose VPA. Based on our results, reducing the dosage of VPA in pediatric patients with epilepsy and adding tPBM therapy can minimize VPA-induced hepatotoxicity and hyperammonemia, with the anticonvulsant efficacy maintained to a certain extent; however, additional basic and clinical studies are necessary to confirm these findings.

Adding tPBM to high-dose VPA exerted adverse effects on PTZ-induced seizures in terms of the maximum seizure stage, the total duration of stage 4–7 seizures, and the latency to stage 6 seizures. Thus according to these results, a low dose of or even a reduction in the VPA dosage should be considered when applying tPBM as an add-on therapy, but the combined use of tPBM with a high dose of VPA directly, without reducing the VPA dosage, should be avoided.

We observed that tPBM could offset the anticonvulsant effect of high-dose VPA; however, its underlying mechanisms remain to be elucidated. The transcranial administration of PBM preserved the perisomatic inhibition of PV-INs in rats with PTZ-induced SE [8]. After the absorption of NIR light by the CcO of PV-INs, CcO enhances ATP production, which increases the synthesis of GABA by PV-INs. Thus, the PV-INs preserved by tPBM release GABA in the postsynaptic GABAARs, a Cl^−^- selective ion channel, of principal cells. Hence, tPBM might indirectly act as a positive modulator of GABAARs by enhancing the presynaptic synthesis of GABA and the postsynaptic binding of GABA to GABAARs in principal cells. Radwan et al. [43] suggested that tPBM mimics the effects of AEDs such as BZD, barbiturates, and baclofen in their blocking effect on GABA_B_ receptors (referring to the effect of tPBM as a positive modulator of GABA_B_ receptors). The chemical convulsant used in this study, PTZ, is a GABA_A_ receptor antagonist [92]. We previously demonstrated that tPBM attenuated PTZ-induced seizures [8]. Therefore, we speculated that tPBM could also act as a positive modulator of the GABAARs of hippocampal principal cells, thus offsetting the negative modulation of PTZ on GABAARs and consequently optimizing the binding of GABA released from PV-INs.

Adding tPBM therapy to VPA might indirectly modulate the GABAARs of principal cells. VPA increases GABA concentration by increasing presynaptic GABA synthesis and inhibiting GABA metabolism [93]. Most of the GABA released after VPA induction release of GABA binds to GABAARs rather than GABA_B_ or GABA_C_ receptors. Two forms of GABAARs exit with different subunit compositions and locations on the cell membrane of principal cells: synaptic GABAARs and extrasynaptic GABAARs. Synaptic GABAARs mediate phasic inhibition by responding to GABA released across the synaptic cleft. Binding to these low-affinity receptors results in a transient and rapidly desensitizing post-synaptic response on the order of milliseconds [94]. By contrast, extrasynaptic GABAARs have a high affinity to GABA and exhibit persistent tonic inhibition [95], which is a long-term inhibition [94]. A homeostatic competition might exist between phasic and tonic inhibition [96].

**Fig. 4:**
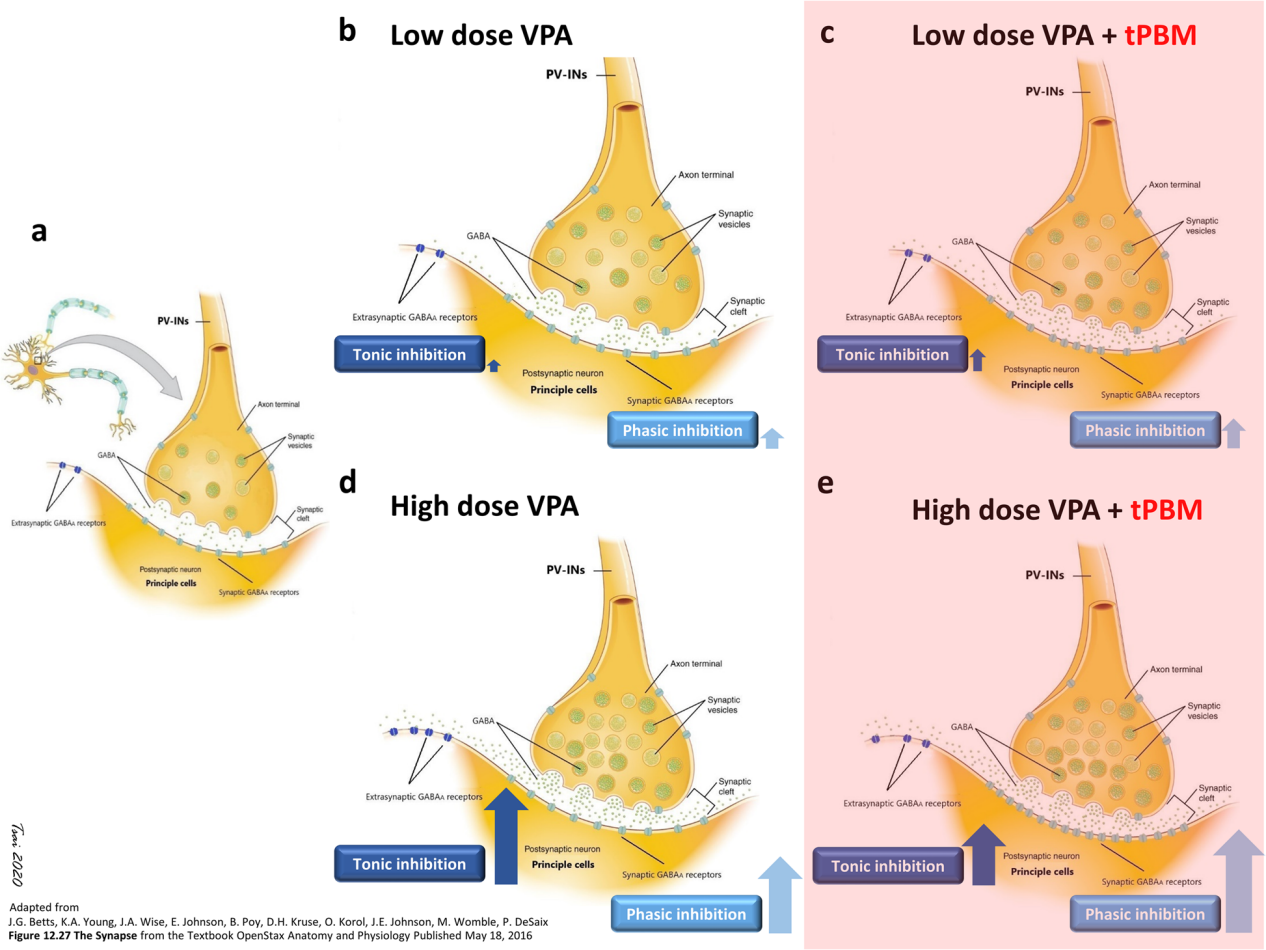
Schematic of the proposed conjecture of GABA_A_ receptors in low-dose VPA with and without add-on tPBM therapy. Dynamic changes in phasic inhibition generated by the binding of GABA (green dots) to synaptic GABAARs (depicted as light blue) and tonic inhibition caused by the binding of GABA to extrasynaptic GABAARs (dark blue) on the postsynaptic neurons of principal cells are shown. The scenarios with add-on tPBM are depicted with a light red background. **a** Without VPA or tPBM treatment, only PTZ was injected, and a baseline amount of GABA was released at the synaptic cleft, with some binding to synaptic GABAARs. **b** When parvalbumin-positive interneurons (PV-INs), the presynaptic neurons, received low-dose VPA, only a limited amount of GABA was bound to synaptic and extrasynaptic GABAARs, thus resulting in limited phasic inhibition (light blue arrow) and tonic inhibition (dark blue arrow). However, phasic inhibition only lasted for milliseconds and was already desensitized upon PTZ injection. **c** In the scenario of low-dose VPA with add-on tPBM, slightly more GABAs was bound to synaptic and extrasynaptic GABAARs after add-on tPBM compared with low-dose VPA without add-on tPBM. Therefore, new phasic inhibition and more tonic inhibition might contribute to the increased attenuation of SE relative to low-dose VPA without add-on tPBM. **d** In the high-dose VPA group, a large amount of GABA was produced and released from the axons of PV-INs. The abundant amount of GABA was not only bound to synaptic GABAARs and caused transient phasic inhibition but also effectively diffused to extrasynaptic GABAARs, thus resulting in considerable tonic inhibition after VPA injection; this long-term inhibition could last after PTZ injection, thus resulting in the longest suppression of SE. **e** Adding tPBM to high-dose VPA increased the preexisting large amount of presynaptic GABA production and release that were stimulated by high-dose VPA injection. A large amount of GABA was bound to synaptic GABAARs, thus resulting in high phasic inhibition, which competes with tonic inhibition and subsequently reduces phasic inhibition. Therefore, the suppression of PTZ-induced SE was inhibited. The use of the material in Fig. 4 was permitted under the Creative Commons Attribution 4.0 International license (https://creativecommons.org/licenses/by/4.0/deed.en) of the “File:1225 Chemical Synapse.jpg” from Wikimedia Commons (the free media repository [https://commons.wikimedia.org/wiki/File:1225_Chemical_Synapse.jpg]). Changes were made, and the original artwork is credited to J.G. Betts, K.A. Young, J.A. Wise, E. Johnson, B. Poe, D.H. Kruse, O. Korol, J.E. Johnson, M. Womble, P. Desaix for their “Figure 12.27 Synapse” from the Textbook OpenStax Anatomy and Physiology, published on May 18, 2016 (source: https://openstax.org/books/anatomy-and-physiology/pages/12-5-communication-between-neurons)

VPA increases GABA release from the axonal terminal of PV-INs and inhibits the reuptake of GABA. Most of the released GABA binds to synaptic GABAARs, and some GABA that escapes presynaptic reuptake [97] binds to the extrasynaptic GABAARs of principal cells. Along with the homeostatic competition between the phasic and tonic inhibition of GABAARs, the following hypothesis is proposed to explain the offsetting effects of adding tPBM to high-dose VPA (Fig. 4). Add-on tPBM might increase the release of GABA rom the axonal terminal of PV-INs at perisomatic inhibitory networks; this speculation is supported by our previous study [8]. In our experimental protocol, tPBM was added 30 min after VPA injection. Thus, in the low-dose VPA scenario (Fig. 4b), the phasic inhibition of synaptic GABAARs could be rapidly desensitized before the addition of tPBM. Therefore, synaptic GABAARs could once again be sensitized through binding with GABA released due to the addition of tPBM (Fig. 4c), with the result being a synergistic anticonvulsant effect facilitating by Cl^−^ influx. However, only a limited amount of GABA that escaped from presynaptic reuptake through the GABA transporter reaches extrasynaptic GABAARs; hence, tonic inhibition was limited. Consequently, the rats receiving tPBM added to low-dose VPA still presented prominent SE. However, medium- and high-dose VPA may induce PV-INs to synthesize a large amount of GABA and cause the universal binding of GABA to its binding sites (GABA site) of both synaptic and extrasynaptic GABAARs, thus resulting in phasic and tonic inhibition, respectively. Long-term tonic inhibition effectively offsets the opposite action of PTZ on the picrotoxin site of GABAARs and eventually suppresses SE. Compared with the rats treated with a low dose of VPA, the rats treated with a high dose of VPA (Fig. 4d) had more extrasynaptic GABAARs that were bound to ambient GABA that strongly suppressed SE. However, compared with adding tPBM to low-dose VPA, tPBM added to high-dose VPA increased the synthesis and release of presynaptic GABA and its bindings to synaptic GABAARs were more than those in synaptic GABAARs with low-dose VPA added-on with tPBM. When increased phasic inhibition occurs in principal cells after treatment with add-on tPBM and high-dose VPA, phasic inhibition might have competed with tonic inhibition [96]. Therefore, low tonic inhibition exacerbates SE compared with high-dose VPA without tPBM (Fig. 4e, the schematic diagram is shown in Fig. 4). The aforementioned hypothesis was based on the findings of this study. Additional studies are required to confirm the hypothesis.

Add-on tPBM might directly modulate GABAARs. Visible and ultraviolet light directly modulate GABAARs [98] and the GABAAR-mediated currents of cortical neurons [99]. We assumed that tPBM with a wavelength of 808 nm directly modulates GABAARs through an unknown mechanism and acts as a positive modulator. Likewise, adding tPBM to low-dose VPA might directly and positively modulate the GABAAR-induced current and enhance phasic inhibition and a portion of tonic inhibition; the addition of tPBM to high-dose VPA might considerably enhance phasic inhibition and suppress tonic inhibition due to homeostatic competition between phasic and tonic inhibition [96].

### Limitations and future work

This study has some limitations. Because this study was performed in a non-blinded manner, it lacked interobserver and intraobserver reliability. A small sample size was included in this study. Therefore, the lower incidence rate of severe SE in the VPA100 + tPBM + PTZ group than in the VPA100 + PTZ group was attributable to only one rat, and this finding was not sufficient to infer that the lower incidence was due to add-on tPBM therapy. Regarding the tPBM protocol, the single irradiation administered in this study may be a limitation because it did not mimic regular tPBM maintenance treatment for CSE prevention. For better bench-to-bed translation of regular tPBM add-on to VPA for CSE prevention, experimental designs involving repetitive tPBM, such as irradiation once daily, should be considered in future studies. Moreover, irradiance at the target in this study was higher than 1 W/cm^2^, which might generate heat over the scalp [100]. Additionally, the thermal effect was not evaluated in this study. However, the photothermal effects of tPBM are less likely to occur in the brain tissue due to poor heat penetration deep into the brain [101], and the superficial heat of the skin and scalp did not affect the therapeutic effect of tPBM due to CcO and cerebral hemodynamics [100]. However, future studies should examine the calibration of thermal effect. Furthermore, the optimal conditions should be determined for use of tPBM as an add-on therapy to VPA or even other AEDs. More convulsive doses for single injection of PTZ model and PTZ kindling model with more time-points of sub-convulsive dose PTZ injection should be also considered to verify the synergistic anticonvulsive effects of tPBM add-on to AEDs in future works. As for VPA, synergistic effect of tPBM was shown when added-up to low-dose VPA. VPA with lower doses than 100 mg/kg should be designed in future works. In this study, we didn’t check safety indicators of add-on therapy of tPBM to VPA including the hepatic and renal function, hemograms including white blood cells and platelets, and these should be included in future works. Future studies should perform histopathological examinations of rat brains, especially the hippocampus; such examinations should include hematoxylin and eosin staining for neuronal damage, TUNEL assay for apoptosis, and propidium iodide staining for necrosis, and electron pathology study for examining mitochondrial morphology. Finally, more future experiments are needed to be designed to demonstrate the synergistic effects of tPBM add-on to AEDs on seizures. To enable the application of tPBM add-on therapy in patients, studies in human cells are warranted. In view of the current progress of epilepsy patient–derived induced pluripotent stem cells platforms for seizure studies [102], additional experiments applying tPBM as an added-on to AEDs in these models should be performed.

## Conclusions

In this study, we demonstrated that VPA pretreatment with tPBM add-on therapy (combination therapy and polytherapy) exerted a significant statistical synergistic effect that delayed the latency to stage 2 seizures. However, tPBM add-on therapy to medium- and high-dose VPA increased the maximum seizure stage and prolonged the total duration of stage 4–7 seizures. In terms of future CSE prevention strategies, the application of tPBM add-on therapy to low-dose rather than medium- or high-dose VPA for pediatric patients, especially pubertal patients with CSE, could allow for a reduction in the VPA dosage, thus minimizing its side effects such as hepatotoxicity while maintaining therapeutic efficacy once an optimal protocol is established.

## Data Availability

The data used and analyzed during the current study are available from the corresponding author.

## Abbreviations

CSE: Convulsive status epilepticus
VPA: Valproic acid
PBM: Photobiomodulation
NIR: Near-infrared
CcO: Cytochrome *c* oxidase
tPBM: Transcranial photobiomodulation
PTZ: Pentylenetetrazole
AEDs: Antiepileptic drugs
BZD: Benzodiazepine
SE: Status epilepticus
sIPSCs: Spontaneous inhibitory postsynaptic currents
GABA: γ-amino butyric acid
GABAARs: GABAA receptors
NMDA: N-methyl-D-aspartate
PV: Parvalbumin
INs: Interneurons
PV-INs: Parvalbumin-positive interneurons
TUNEL: Terminal deoxynucleotidyl transferase dUTP nick end labeling
LDR: Low-dose gamma irradiation
LLLT: Low-level laser (light) therapy
GTCS: Generalized tonic–clonic seizures
WDS: Wet-dog shaking
